# Supplementary material for: A high-resolution mRNA expression time course of embryonic development in zebrafish
Source: eLife. 2017 Nov 16;6:e30860. doi: 10.7554/eLife.30860 (PMC5690287; doi:10.7554/eLife.30860)
Supplement: Supplementary file 6. [file elife-30860-supp6.zip › biolayout-clusters-files/Cluster084-genes.html]

Cluster084


# Cluster084: Genes

| Ensembl ID | Gene Name | Chr | Start | End | Biotype |
| --- | --- | --- | --- | --- | --- |
| ENSDARG00000006666 | BUD13 | 21 | 22660454 | 22668742 | protein\_coding |
| ENSDARG00000102154 | BX855614.3 | 4 | 72116419 | 72132500 | protein\_coding |
| ENSDARG00000074270 | ENSDARG00000074270 | 12 | 29108376 | 29118836 | protein\_coding |
| ENSDARG00000063051 | bap1 | 22 | 10562276 | 10574844 | protein\_coding |
| ENSDARG00000061400 | csgalnact2 | 13 | 25439381 | 25450853 | protein\_coding |
| ENSDARG00000021945 | ddx23 | 23 | 14437470 | 14460429 | protein\_coding |
| ENSDARG00000099739 | ddx41.1 | KN150642.1 | 42452 | 55111 | protein\_coding |
| ENSDARG00000062749 | ino80b | 7 | 7463296 | 7473849 | protein\_coding |
| ENSDARG00000104484 | itgb1b | KN149986.1 | 624 | 7222 | protein\_coding |
| ENSDARG00000068698 | psenen | 15 | 37644270 | 37648753 | protein\_coding |
| ENSDARG00000095510 | si:ch211-10d23.5 | 23 | 4335420 | 4342160 | protein\_coding |
| ENSDARG00000101029 | si:ch73-347e22.4 | 19 | 10941560 | 10953544 | protein\_coding |
| ENSDARG00000042724 | supt5h | 15 | 20428863 | 20459520 | protein\_coding |
| ENSDARG00000027397 | vangl2 | 7 | 5202345 | 5253498 | protein\_coding |
| ENSDARG00000074453 | zfx | 24 | 16861228 | 16877034 | protein\_coding |
